# Consensus on Current Injectable Treatment Strategies in the Asian Face

**DOI:** 10.1007/s00266-016-0608-y

**Published:** 2016-02-18

**Authors:** Woffles T. L. Wu, Steven Liew, Henry H. Chan, Wilson W. S. Ho, Nantapat Supapannachart, Hong-Ki Lee, Adri Prasetyo, Jonathan Nevin Yu, John D. Rogers, Asian Facial Aesthetics Expert Consensus Group

**Affiliations:** Camden Medical Centre, Woffles Wu Aesthetic Surgery and Laser Centre, 1 Orchard Boulevard, Suite #09-02, Singapore, 249615 Singapore; Shape Clinic, Sydney, Australia; Department of Medicine, University of Hong Kong, Pokfulam, Hong Kong; The Specialists: Lasers, Aesthetic and Plastic Surgery Central, Pokfulam, Hong Kong; Department of Dermatology, Ramathibodi Hospital, Mahidol University, Bangkok, Thailand; Image Plastic Surgery Clinic, Seoul, Korea; REJUVA Clinic, Surabaya, Indonesia; JY Dermatology and Aesthetic Center, Manila, Philippines; Regional Medical Affairs, Allergan Asia Pacific, Singapore, Singapore

**Keywords:** Facial esthetics, Esthetic outcomes, Treatment strategies, Hyaluronic acid fillers, Botulinum toxin, Aging

## Abstract

**Background:**

The desire for and use of nonsurgical injectable esthetic facial treatments are increasing in Asia. The structural and anatomical features specific to the Asian face, and differences from Western populations in facial aging, necessitate unique esthetic treatment strategies, but published recommendations and clinical evidence for injectable treatments in Asians are scarce.

**Method:**

The Asian Facial Aesthetics Expert Consensus Group met to discuss current practices and consensus opinions on the cosmetic use of botulinum toxin and hyaluronic acid (HA) fillers, alone and in combination, for facial applications in Southeastern and Eastern Asians. Consensus opinions and statements on treatment aims and current practice were developed following discussions regarding pre-meeting and meeting survey outcomes, peer-reviewed literature, and the experts’ clinical experience.

**Results:**

The indications and patterns of use of injectable treatments vary among patients of different ages, and among Asian countries. The combination use of botulinum toxin and fillers increases as patients age. Treatment aims in Asians and current practice regarding the use of botulinum toxin and HA fillers in the upper, middle, and lower face of patients aged 18 to >55 years are presented.

**Conclusions:**

In younger Asian patients, addressing proportion and structural features and deficiencies are important to achieve desired esthetic outcomes. In older patients, maintaining facial structure and volume and addressing lines and folds are essential to reduce the appearance of aging. This paper provides guidance on treatment strategies to address the complex esthetic requirements in Asian patients of all ages.

**Level of Evidence V:**

This journal requires that the authors assign a level of evidence to each article. For a full description of these Evidence-Based Medicine ratings, please refer to the Table of Contents or the online Instructions to Authors www.springer.com/00266.

## Introduction

The advent of safe and predictable injectable agents, expanded indications for their use, and improved understanding of their role in facial volumization, has helped practitioners advance from ‘two-dimensional’ treatment strategies previously used to reduce facial lines in aging, toward the use of injectable treatments aimed at creating three-dimensionality to achieve facial esthetic restructuring and rejuvenation in patients of all ages. These three-dimensional changes were previously only attainable through surgery, but now can be achieved with botulinum toxin and hyaluronic acid (HA) fillers in many situations.

In Asia, as in Western countries, botulinum toxin is used extensively to treat dynamic lines in the upper face [1]. Botulinum toxin is also used to reshape and recontour the face to correct perceived undesirable anatomical features (e.g., reduce bulk of the masseter and activity of the mentalis) in younger patients, and for rejuvenation in older patients [1–9]. Intradermal injection of minute doses of botulinum toxin into the full face and neck to achieve skin rejuvenation [4, 9–11] has also gained popularity in some parts of Asia.

**Fig. 1:**
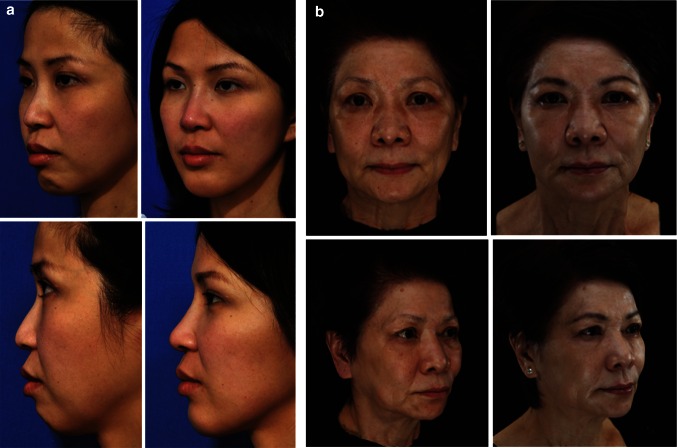
Effects of combination treatment in **a** younger and **b** older Asian patients. **a** A 38-year-old Asian woman with a retruded supraorbital rim, low radix and nasal dorsum, retruded medial malar, recessed nasal spine, and hyperactive mentalis muscle, before treatment (*left panels*). An esthetically improved facial appearance (*right panels*) was achieved after dermal fillers were used to augment the supraorbital rim, the nasal dorsum, nasal spine, columella, and the medial cheek, and botulinum toxin and HA fillers were used to improve chin projection and relax the hyperactive mentalis muscle as follows: BOTOX^®^ (Allergan, Inc.), 16 U to mentalis; Juvéderm^®^ Voluma™ (Allergan, Inc.), 1.5 ml to both medial cheeks, 1.2 ml to the nose, 1.2 ml to the chin; Juvéderm^®^ Ultra Plus (Allergan, Inc.), 0.4 ml to supraorbital rim. (Photos courtesy of Dr. Steven Liew). **b** A 65-year-old Asian woman showing pan-facial aging, particularly tissue deflation and volume loss in the temple and periocular region, the perioral region, and the rest of the lower face, before treatment (*left panels*). A significant improvement in appearance and youthfulness was achieved following combination therapy (*right panels*), in which botulinum toxin was used in the upper face to reshape her eyebrows; and HA filler was used to volumize and restructure the temple, the tear trough, lateral cheek, nasolabial fold, perioral region, jawline, and chin as follows: BOTOX^®^, 24 U in the upper face, 6 U in the mentalis, 30 U in the jawline and platysma; Juvéderm^®^ Voluma™, 1.2 ml in temples, 1 ml in both cheeks, 0.8 ml in pre-auricular area, 1 ml in marionette lines and jowls; Juvéderm^®^ Volift™ (Allergan, Inc.), 1 ml in supraorbital rims, 1.6 ml in lips, 0.4 ml in nasolabial folds; Juvéderm^®^ Volbella™ (Allergan, Inc.), 1 ml in both tear troughs (Photos courtesy of Dr. Peter Peng)

HA fillers are used more commonly and extensively for facial restructuring purposes in young Asians than in young Western patients, to address structural features and deficiencies that are perceived as unesthetic [12]. Physicians commonly use HA to reshape and recontour facial features, to address structural deficiency in the projection of the midline facial features (medial malar, forehead, nose, chin, and glabella), to correct “flatness” of the infraorbital and medial malar regions, to reduce under-eye shadows, and to improve projection and definition of the nose and chin, thereby creating a visual illusion of “narrowing” to the whole face (Fig. 1a) [9, 13–19]. More recently, physicians have used HA to volumize and reshape the Asian forehead to create an esthetically pleasing, convex, and youthful contour.

For older Asians, fillers are mainly used to restore volume loss in areas of the face that show signs of aging due to volume deflation and tissue descent (such as the brow, midface, periorbital, pre-auricular, and perioral regions), and to address areas of intrinsic anatomical structural deficiency that become more pronounced with age-related volume loss, such as the medial malar, nose, inferior orbital rim, jawline, pre- and post-jowl regions, perialar recess, and chin (Fig. 1b).

Botulinum toxin and HA fillers are often used in combination to achieve the esthetic outcomes desired by Asians (Fig. 1). Combined treatment results in synergistic improvement compared to individual use of these agents and is used as “age-prevention” treatment in younger patients [20]. Although numerous papers describing these techniques in Western populations have been published [20–24], only one English-language paper that describes combination injectable treatment in Asians could be found in PubMed [25].

The physical features characteristic of the Asian face are related to specific skeletal and morphological features that differ from those of Caucasians [12]. Hence, different treatment strategies are required to achieve esthetic or anti-aging outcomes in Asians. To address the lack of adequate recommendations and published clinical evidence available to physicians who treat Asian patients for facial esthetic concerns, the Asian Facial Aesthetics Expert Consensus Group, comprising eight plastic surgeons, 11 dermatologists, an esthetic physician, and an anatomist from 11 Asia Pacific countries (Table 1), met in Seoul on June 4–5, 2014 to discuss current local practices. The physicians had 7–30 years’ (mean 17 years) experience in facial esthetics [12]. These experts were chosen based on their vast experience of treating Asian patients, their presence on advisory boards, the frequency of their presentations at regional and international conferences, and their contribution to the literature. Described here are their consensus opinions regarding the cosmetic facial use of botulinum toxin and HA fillers in Asians from Southeast and Eastern Asia, alone and in combination.

**Table 1:** Members of the Asian Facial Aesthetics Expert Consensus Group

Member	Specialty	Practice	Years of experience in esthetic practice	Country
Dr. Greg Goodman	Dermatologist	Private	30	Australia
Dr. Woffles Wu	Plastic surgeon	Private	25	Singapore
Dr. Hee Jin Kim	Anatomist	Public/university	23	Korea
Dr. Nantapat Supapannachart	Dermatologist	Public/university hospital	20	Thailand
Dr. Hideaki Sato	Plastic surgeon	Public/university hospital	20	Japan
Dr. Taro Kono	Plastic surgeon	Public/university hospital	20	Japan
Dr. Nobutaka Furuyama	Plastic surgeon	Private/university hospital	20	Japan
Dr. Henry Chan	Dermatologist	Private/university hospital	19	Hong Kong
Dr. Wilson Ho	Plastic surgeon	Private/university hospital	16	Hong Kong
Dr. Jonathan Yu	Dermatologist	Private	16	Philippines
Dr. Danru Wang	Plastic surgeon	Public/university hospital	16	China
Dr. Steven Liew	Plastic surgeon	Private	15	Australia
Dr. Hong Ki Lee	Plastic surgeon	Private	15	Korea
Dr. Hsien-Li Peter Peng	Dermatologist	Private	15	Taiwan
Dr. Yan Wu	Dermatologist	Public/university hospital	13	China
Dr. Rashmi Shetty	Esthetic physician	Private	13	India
Dr. Chytra Anand	Dermatologist	Private	13	India
Dr. Vandana Chatrath	Dermatologist	Private	12	India
Dr. Akiko Imaizumi	Dermatologist	Private	12	Japan
Dr. Marisa Pongprutthipan	Dermatologist	Public/university hospital	10	Thailand
Dr. Adri Prasetyo	Dermatologist	Private	7	Indonesia

## Methods of Consensus Development

To determine which noninvasive facial esthetic treatments are most commonly used in Asian patients of different age groups, the Expert Group members completed a pre-meeting online survey and then during the meeting, they were again surveyed to determine (i) the percentage of patients that they treat for each individual indication, based on the total number of patients who present to them for noninvasive facial esthetic treatment, and (ii) the treatment methods they use for each indication. The purpose of the meeting survey was to give an indication of how common each treatment concern is among their Asian patients, and to provide an overview of common practice patterns. Responses from the three Indian experts who attended were not included in the data presented here because treatment of patients from the Indian subcontinent is beyond the scope of this publication. Rare procedures and high-risk treatments requiring extensive experience were also not discussed at the meeting or mentioned in this review.

A PubMed review of the English-language medical literature conducted with the search terms “hyaluronic acid filler,” “botulinum,” “face,” “facial,” “Asia,” “Asian,” “Korea,” “Korean,” “China,” “Chinese,” “Thailand,” “Singapore,” “Indonesia,” “Japan,” and “Philippines” was completed before the meeting. Due to the paucity of prospective, randomized, comparative, controlled studies of botulinum toxin and HA fillers in Asian patients (with only one English-language paper on their combination use in Asians [25] being found), no formal process of evaluating the published clinical evidence could be conducted. The process used to develop the consensus statements presented here has been described [12].

This article does not contain any studies with human participants or animals performed by any of the authors.

## Results: Current Practice and Consensus Opinions

The following content reflects the proceedings of the Asian Facial Aesthetics Expert Consensus Group meeting. The consensus statements presented here were agreed by all the Expert Group members.

### Aims of Treatment in Asians

The aims of facial esthetic treatment in Asians are to achieve clear, youthful, and fair skin; an oval facial shape with smooth facial contours; large expressive eyes with clearly defined palpebral creases; and an esthetically pleasing, structural definition of the nose, chin, eyebrows, and cheeks [12]. To achieve the ideal oval in Asian patients, treatments to narrow and lengthen the lower face may be required. This often involves reducing the bulk of the masseter muscle mass with botulinum toxin, and the use of HA fillers to project and lengthen the chin. Anterior projection of the forehead, brows, and midface (medial cheeks and nose) is a common goal, although the importance of this three-dimensional projection to the overall esthetic outcome may be under-recognized by the patient.

As the average Asian face presents specific esthetic challenges such as a square boxy face shape with lack of vertical height, lack of anterior projection, a flat broad nose, eyebags with prominent infraorbital hollows, retrusive maxilla and chin, deep nasolabial folds, and an obtuse cervicomental angle, it is seldom possible to confine treatment to a specific site. Augmenting the nose with an HA filler automatically places emphasis on the adequacy of chin projection, infraorbital volume, and malar projection, all of which combine to give the face a more esthetic three-dimensionality. At the same time, botulinum toxins may be required to shape the eyebrows, relax mentalis strain, and reduce the volume of hypertrophic masseter muscles, which in turn narrows the width of the lower face and triangulates it [8, 9].

Modern treatment plans thus require attention to surface, volume, and movement in all facial areas. When treating aging patients, the focus of treatment should involve the whole face, rather than merely certain aspects, because treatment of just one feature will affect the overall balance and proportions of the face. To maintain balance and harmony, treatment of different areas of the face must be addressed in a logical sequential fashion, and a global approach can often provide optimal results [4].

It became apparent during this consensus meeting that the significant change in esthetic.

Asian facial concepts and trends over the past four decades have been the realization that Asian patients do not want to look Western. Asian patients aspire to look more beautiful or attractive within their own ethnic esthetic boundaries. Some may desire a Pan-Asian (Eurasian) look, but none wished to look ‘Western,’ in contrast to a common misperception held by many Western doctors. These patients wish to look like good-looking Asians, rather than good-looking Caucasians [8].

### Consensus Statements on Aims of Treatment in Asians

- The aims of esthetic facial treatment in Asians are clear, youthful, fair skin and a smooth, oval facial shape with adequately projected midline features.
- Increasing anterior projection of the midface/forehead and vertical lengthening of facial height are key components of achieving esthetic balance.
- Narrowing the lower face is the key to achieving the ideal oval face shape.
- A full-face approach is required to achieve a balanced, harmonious, more three-dimensional facial appearance.
- Full-face facial enhancement using botulinum toxin and HA fillers is aimed at achieving maximal beauty while maintaining the unique Asian appearance.
- Asian patients do not wish to look ‘Western.’

### Current Practice for Nonsurgical Facial Esthetic Treatment in Asians

The results of the surveys taken by the Expert Group are presented in Tables 2, 3, 4, 5, 6, 7, and 8. The ranges of values listed in Tables 3, 4, 5, 6, 7, and 8 include the responses regarding Asian patients that were given by the experts from China, Hong Kong, Indonesia, Japan, Korea, the Philippines, Singapore, Taiwan, Thailand, and Australia. When stating the range of botulinum toxin units they used, the experts were asked to cite onabotulinum toxin A (Botox^®^; Allergan) units for the sake of uniformity and consistency. To provide guidance regarding the most common responses where practices differed (e.g., between countries), smaller ranges that reflect the majority of the participants’ responses are shown where applicable.

**Table 2:** Summary of the most commonly used noninvasive esthetic treatments in Asian facial esthetics patients according to age

Priority	Patient’s age			
	18–30 years	31–40 years	41–55 years	>55 years
1	Laser/IPL	Laser/IPL/botulinum toxin for facial lines	Botulinum toxin for facial lines	Combination of botulinum toxin and HA fillers
2	Botulinum toxin for issues other than facial lines^a^	Combination of botulinum toxin and HA fillers	Combination of botulinum toxin and HA fillers	HA fillers
3	Botulinum toxin for facial lines/HA fillers	HA fillers	HA fillers/laser/IPL	Laser/IPL/botulinum toxin for facial lines

Based on results of a pre-meeting survey of the Asian Facial Aesthetics Expert Consensus Group

This table shows that younger Asian patients are more concerned about the color and texture of their skin as evidenced by their prioritization of lasers and IPL as their first choice of cosmetic treatments. In the 18–30-year age group, botulinum toxin was used mainly for reducing masseteric bulk, while HA fillers were used mainly for correcting structural deficiencies such as a low nasal bridge or retrusive chin. In the above 55-year age group, this sequence of priorities was reversed

*HA* hyaluronic acid, *IPL* intense pulsed light

^a^For example, masseter hypertrophy, mentalis hyperactivity

**Table 3:** Current practice regarding injectable treatments to address upper face lines and folds in Asians

	Glabella		Forehead	Crow’s feet
Dynamic lines				
Botulinum toxin dosage^a^	Dose	Injection points	Dose	Dose (per side)
	12–20 U (88 % use 15–20 U)	3–10 points to address frowning (for the purpose of movement only, not brow shaping) 88 % use ≥5 injection points	5–12 U (depends on the number of injection points and the dose used at each point, e.g., 1–4 U per point)	6–12 U (depends on the number of injection points and the dose used at each point, e.g., 1–4 U per point)
Static lines				
Percentage of Expert Group using a combination of botulinum toxin and HA fillers, or botulinum toxin only	Combination (100 %)		95 % use combination 5 % use botulinum toxin only	Botulinum toxin only (100 %)

Based on Expert Consensus Group Meeting survey outcomes

In the glabella region, 88 % of injectors use five or more injection points to reduce frowning as well as shape the brow. All injectors use a combination of botulinum toxin and fillers here. In contrast, crow’s feet were only treated with botulinum toxin using a range of 6–12 U per side

*HA* hyaluronic acid

^a^Onabotulinum toxin A units were specified for the sake of uniformity

**Table 4:** Proportion of Asian patients treated for volume deficit in the upper face and volumizing treatment used

	Forehead	Glabella	Eyebrow (filling of lateral and superior orbital rim)	Temple
Percentage of patients^a^	<5–20 % (88 % responded <5–10 %)	<5–20 % (ranges were widely distributed)	5–20 % (88 % responded <5–10 %)	10–35 % (60 % responded 20–30 %)
Percentage of Expert Group using a combination of botulinum toxin and HA fillers, or HA fillers only	88 % combination	100 % combination	81 % combination	100 % HA fillers only

Based on Expert Consensus Group Meeting survey outcomes. Injection technique and dosage of fillers were not discussed because this depends on the product used. Upper eyelids were not included because volumizing treatment of this area is uncommon

Volumization of the glabellar and forehead regions and the lateral and superior orbital rims was largely performed in combination with botulinum toxins to decrease associated wrinkles, whereas in the temples these are treated with fillers only

*HA* hyaluronic acid

^a^Percentage of Expert Group members’ injectable facial esthetics patients

**Table 5:** Proportion of Asian patients treated for middle face lines or reshaping, and botulinum toxin dose used

Bunny lines		Dynamic lines under eyelid		Nasal shape		Gummy smile	
% patients^a^	Total dose^b^	% patients^a^	Total dose^b^	% patients^a^	Total dose^b^	% patients^a^	Total dose^b^
<10–50 % (ranges were widely distributed)	2–10 U (94 % use ranges that include 4– 6 U)	20–40 % (ranges were widely distributed)	4–12 U (88 % use 4–6 U)	0–25 %^c^ (88 % responded ≤5 %)	Lift nasal tip: 2–4 U Alar (flare): 2–8 U	<10 % (81 % stated ≤5 %)	2–12 U, depending on severity (69 % use 4 U or a range that includes 4 U)

Based on Expert Consensus Group Meeting survey outcomes

Dynamic lines under the eyes and bunny lines were the most frequently treated areas in the midface, with dosages ranging from 4 to 6 U for both sides. Treatment of the nose with onabotulinum toxin, either to reduce alar flare or raise the tip by decreasing the action of the depressor muscles, was performed only by some experts

^a^Percentage of Expert Group members’ injectable facial esthetics patients

^b^Onabotulinum toxin A units were specified for the sake of uniformity

^c^To lift the tip or to address alar flare, several experts only treat either flare or tip, but not both

**Table 6:** Proportion of Asian patients treated for volume deficit in the middle third of the face

Age group	Location/indication						
	Cheek complex			Tear trough/lower orbital rim	Nasolabial fold	Nose (any aspect)	Alar base/pyriform margin
	Medial cheek	Lateral cheek	Subzygomatic hollow				
Percentage of patients aged <30 years^a^	<5–40 % (ranges were widely distributed)	0–20 % (75 % responded 0–10 %)	0–15 % (75 % responded 0–5 %)	0–50 % (75 % responded 0–30 %)	0–30 % (69 % responded 0–15 %)	10–60 % (88 % responded 20–40 %)	<5–40 % (94 % responded 5–20 %)
Percentage of patients aged >55 years^a^	10–60 % (81 % responded 30–50 %)	<5–40 % (ranges were widely distributed)	10–50 % (ranges were widely distributed)	15–50 % (ranges were widely distributed)	<5–70 % (ranges were widely distributed)	<5–45 % (88 % responded 5–20 %)	5–60 % (ranges were widely distributed)

Based on Expert Consensus Group Meeting survey outcomes

The most frequently filled areas in the under 30-year age group were the nose, tear trough, and medial cheek areas. In the over 55-year age group, the most frequently filled areas were the nasolabial fold, the medial cheek followed by the tear trough, and subzygomatic hollows

^a^Percentage of Expert Group members’ injectable facial esthetics patients

**Table 7:** Proportion of Asian patients treated for lines and folds of the lower face, and treatment used

Variable	Location/indication					
	Perioral lines	Mentalis	Depressor anguli oris	Masseter	Platysma and/or neck lines	Intradermal botulinum toxin
Dynamic lines						
Percentage of patients^a^	<5–30 % (81 % responded ≤10 %)	5–60 % (ranges were widely distributed)	<5–40 % (ranges were widely distributed)	10–50 % (ranges were widely distributed)	5–60 % (ranges were widely distributed)	0–40 % (75 % responded 0–10 %)
Botulinum toxin^b^ dosage	2–8 U (75 % use a range that included 4 U)	2–20 U (56 % use 4–8 U)	2–8 U (74 % said a range that included 4–6 U)	20–100 U (81 % use a range that included 40–80 U)	10–80 U (69 % use 10–30 U)	10–50 U (ranges were widely distributed)
Static lines						
Percentage of Expert Group using combination treatment (C) or botulinum toxin (B) or HA fillers (F) alone	69 % C 25 % B 6 % F	69 % B 31 % C	60 % B 40 %C	100 % B	87 % B 13 % C	100 % B

Based on Expert Consensus Group Meeting survey outcomes

The mentalis and the platysma were the most frequently treated areas with botulinum toxin; the mentalis usually in combination with an HA filler. Reduction of masseteric hypertrophy was the third most frequently treated area of the lower face with botulinum toxin, with a range of 40–80 U used for both sides

^a^Percentage of Expert Group members’ injectable facial esthetics patients

^b^Onabotulinum toxin A units were specified for the sake of uniformity

**Table 8:** Proportion of Asian patients treated for volume/structural deficits in the lower face, and the type of treatment used

Variable	Location/indication			
	Lips	Marionette/pre-jowl sulcus	Chin	Jawline
Percentage of patients^a^	5–30 % (81 % said 10–20 %)	10–50 % (ranges were widely distributed)	10–60 % (88 % said 20–40 %)	5–40 % (81 % said 10–30 %)
HA fillers (F) only or combination (C) treatment with botulinum toxin and HA fillers	63 % F 37 % C	81 % C 16 % F	100 % C	75 % C 25 % F

Based on Expert Consensus Group Meeting survey outcomes

All experts responded that the chin was always treated with a combination of HA filler and botulinum toxin in order to achieve better chin projection and elongation free of any signs of mentalis strain

*HA* hyaluronic acid

^a^Percentage of Expert Group members’ injectable facial esthetics patients

### Most Common Treatments According to Patient Age

**Fig. 2:**
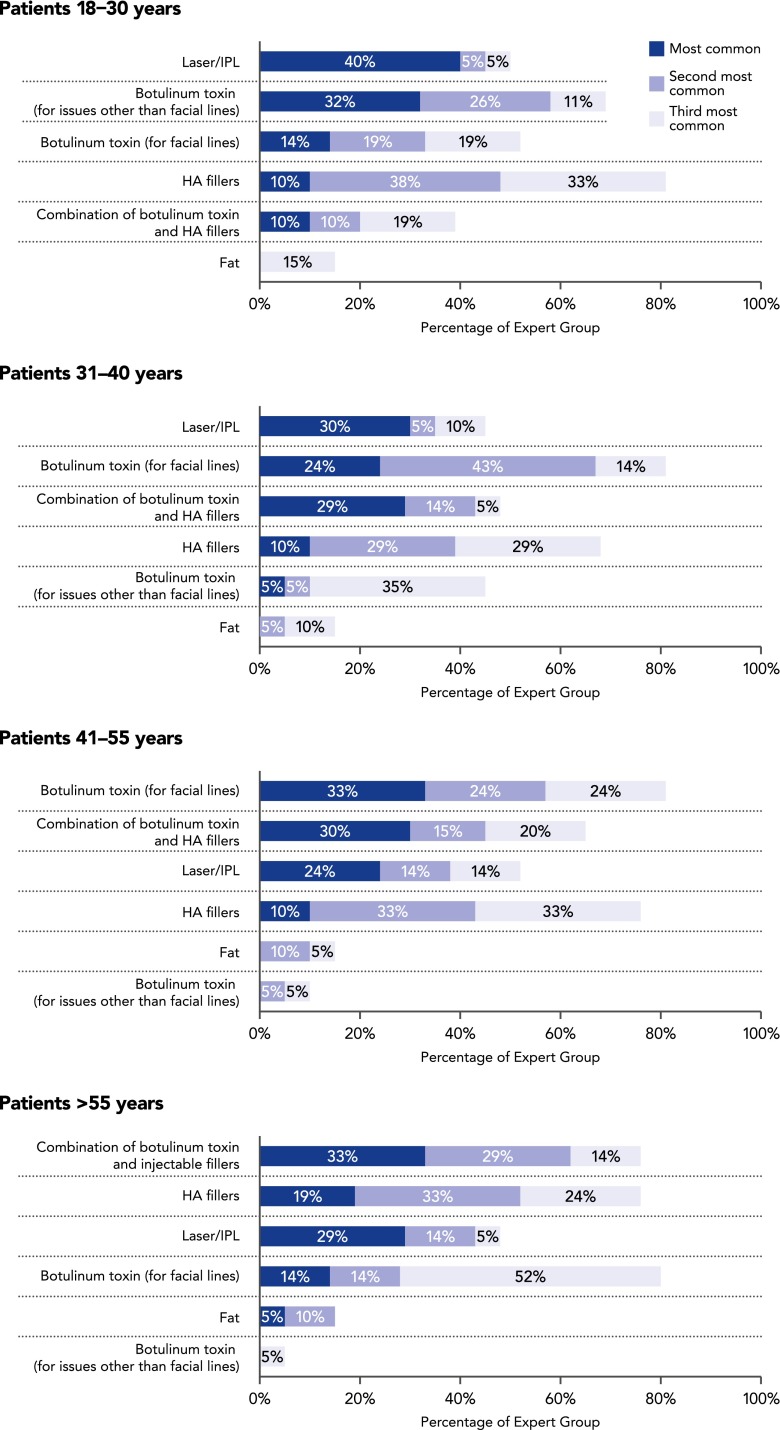
Nonsurgical treatments most commonly used by the Expert Group for their Asian facial esthetics patients, according to patient age. Based on results of a pre-meeting survey of the Asian Facial Aesthetics Expert Consensus Group. *HA* hyaluronic acid, *IPL* intense pulsed light

The experts’ responses regarding the most commonly used nonsurgical facial treatments in their Asian patients are shown in Fig. 2 and Table 2. The survey revealed that lasers/intense pulsed light are the most common treatments used in Asian women younger than 40 years, most likely because improvement of skin tone, color, texture, and clarity is universally desirable. Skin treatments are not described further because they are beyond the scope of this manuscript. The survey also showed that the use of botulinum toxin and fillers, alone and in combination, is very common in Asians (Fig. 2). Patterns of use vary among patients of different ages, according to the changes that result from facial aging and volume depletion, with combination use of botulinum toxin and fillers increasing as patients age. In young Asian patients wanting to increase their attractiveness, treatment invariably involves increasing the vertical height and anterior projection of the face with fillers, and reducing lower facial width with botulinum toxin. Older patients request facial rejuvenation procedures to address age-related changes that are compounded by anatomical structural deficiencies [12].

### Treatment Areas, Indications, and Dosage

#### Upper Face

Being asked which are the most common types of dynamic lines observed in the upper face of their Asian patients, 69 % (11/16) of the experts responded that it is the crow’s feet indication that is most commonly treated, while 31 % (5/16) mentioned glabella lines. The treatment used by these experts to address upper facial lines is summarized in Table 3.

Botulinum toxin doses used by this group of experts to treat horizontal lines on the forehead are generally lower than those used in Caucasians [26, 27] and in guidelines published by the Korean Academy of Corrective Dermatology [1]. Suggested reasons were as follows: Asians prefer a more natural-looking appearance (i.e., preservation of facial expression) for the forehead and often have fewer wrinkles in the upper face compared with age-matched Caucasians [28]. There is less use of muscles in facial expression and communication in the forehead area in Asians, compared with Caucasians [29]. Finally, Asians tend to have the appearance of “puffy” eyelids, and any inadvertent eyebrow ptosis and heaviness of the brow will exacerbate eyelid heaviness. Overtreatment and isolated treatment of the frontalis (without co-treatment of the glabella) is to be avoided. The dosage used in the forehead varies according to the reason for treatment (e.g., to address lines in the forehead, elevate the lateral brow, or smoothen the forehead texture). The experts agreed that this relatively lower dosage in the forehead and the resulting shorter duration of effect are acceptable to their patients. To ensure a more natural appearance, patients accept that they must return for treatment more frequently.

The general consensus regarding the number of glabella injection points was at least five points, depending on the clinical presentation at the time of injection. The majority of experts (88 %) use 15–20 U onabotulinum toxin A in total. This is similar to, or slightly lower than, consensus recommendations of 5–30 U in Caucasian women [26, 27]. A lower dose in the glabella reduces the risk of splayed eyebrows, avoids medial brow ptosis, achieves a more natural-looking result, and preserves more normal movement of the muscles. The number of injection points for crow’s feet lines was not specified because of the wide range of techniques and the wide range of botulinum toxin dosage used.

The proportion of Asian patients who are treated for volume deficit in the upper face, and the treatment types used by these experts, is shown in Table 4. Volumization and contouring of the upper third of the face in Asians has been a well-established indication over the past 5 years among the members of the Expert Group, in contrast to the West, where facial volumization with HA fillers has only more recently gained popularity [20].

Most Asians prefer a smooth forehead and glabella, and full temples without hollows. In Korea, younger women are more commonly treated to create a smooth, convex forehead than those in other parts of Asia. Glabella volumization is common in elderly East Asians (Chinese, Japanese, Koreans). Age-related volume loss compounds the effect of a flat, retruded frontal bone, which creates an obvious flattening of the glabella that is considered undesirable by many Asian women. Most experts agreed that there is a need for more volumization and shaping of the upper face for many of the Asian patients who request facial esthetic treatment. The forehead, temple, and glabella are the most commonly volumized areas. For volumization of the forehead and glabella, botulinum toxin is used in combination with fillers to minimize activity of the glabella and frontalis, and facilitate the integration of the filler into the tissue. The Expert Group highlighted that injecting the glabella is associated with a risk of vascular complications and should remain the domain of advanced practitioners.

Using fillers on the superior orbital rim improves the forward projection of the supraorbital ridge and reduces the appearance of “puffy” eyes. Although patients seldom request this treatment, these experts recommend it to achieve this outcome.

### Consensus Statements on Upper Face Treatment

- In Asia, most patients prefer a natural look, with a smooth forehead that retains the ability to show facial expression.
- The relatively lower botulinum toxin dosages used in the forehead, and the associated potential shorter duration of effect, are acceptable to Asian patients.
- Upper face volumization is common among Asian patients, to achieve optimal smoothness and anterior projection of the forehead and upper face.
- In general, younger women want a smooth forehead, whereas older women want a smooth, contoured forehead without temporal hollows.

#### Middle Face

The use of botulinum toxin to address dynamic lines in the middle face, nasal shape, and gummy smile, and the proportion of Asian esthetic patients treated for each indication, is summarized in Table 5. Dynamic lines under the eye can result from muscle activity (facial expression) and/or volume deficiency (inadequate structural support for skin and tissue). The proportion of patients seeking treatment to address infraorbital and bunny lines is relatively high in some countries, although Asians are less prone to photoaging (those younger than 60 years generally have fewer facial wrinkles than age-matched Caucasians [28, 30, 31]). Any lines that become more noticeable are a concern for Asians. The proportion of patients seeking treatment may also reflect a lower tolerance of expression lines by Asians than Caucasians.

With its lack of dorsal height, poor tip support, and wide nasal alar base, the Asian nose can often be treated with HA fillers and botulinum toxin rather than surgery [25, 32]. Gummy smile can be caused by bony structural, dental, or gingival factors, or muscle hyperactivity, all of which may occur alone or in combination [33]. Bimaxillary protrusion is a typical anatomical feature in Asians, which may also explain the common need to treat gummy smile and to relieve mentalis strain.

**Fig. 3:**
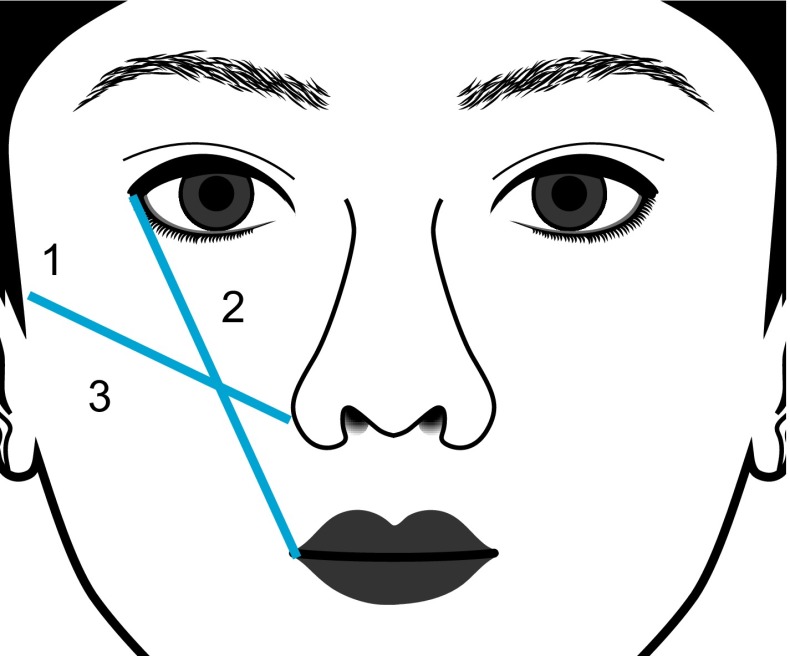
Locations of midface malar volumization. *1* Lateral cheek; *2* medial cheek; *3* subzygomatic hollow

The proportion of Asian patients who are treated with HA fillers for volume deficit in the midface is shown in Table 5. Figure 3 shows the subregions treated for malar volumization described in Table 6. In Asian patients, HA fillers are most frequently used in the middle third of the face. Infraorbital and mid-cheek volume loss are very common and may occur as early as the second decade in Asians, giving the impression of tiredness. The desire to address the perceived undesirable facial features that correlate with underlying characteristic anatomical structures [12] accounts for the relatively high proportion of young patients seeking treatment to volumize the cheek area and alar base, and to address nasal shape. It is important to note that HA fillers are preferentially placed in the medial cheek, and rarely in the lateral part, which could cause widening of an already wide Asian midface. This is in contrast to treatment of Caucasians, where HA fillers are placed laterally to create cheekbones and widen the face.

In Asians, the resulting increased anterior projection of the medial cheek and other midline structures provides a visual narrowing effect, which is a more desirable esthetic outcome. The greater proportion of older patients who receive volumization treatment (Table 6) reflects increasing midface volume loss with aging, which is compounded by the aforementioned structural deficiencies. The use of HA fillers to treat nasolabial folds in Chinese and Korean patients has been described elsewhere [13, 34]. Among these experts, the common approach is first to address the midface, with subsequent treatment directed at the nasolabial folds, should it be necessary.

### Consensus Statements on Midface Treatment

- Because Asians are less prone to photoaging [28, 30, 31], they generally have fewer wrinkles than age-matched Caucasians. Hence, fine lines such as bunny lines and dynamic lines under the eye become a concern and lead to requests for treatment among Asians.
- In younger Asian patients, the main treatment requirements are structural support for an underdeveloped medial cheek, and optimization of nasal shape and chin contour; the medial cheek is the most common area for volumization.
- In older Asian patients, the main focus of treatment is to restore volume of the midfacial areas associated with age-related volume loss.

#### Lower Face

The proportion of Asian patients who receive injectable treatment for lines and folds of the lower face, and the treatments used, is shown in Table 7. Treatment for perioral wrinkles is less common in Asia than in Western countries [1]. The botulinum toxin dosage used by most of these experts to address perioral lines and the depressor anguli is similar to the dosing recommendations for Caucasians (4–6 and 2–15 U, respectively), but is lower for platysmal bands, being 10–30 U by most experts (30–60 U are recommended in Caucasians) [27]. A hyperactive mentalis associated with retro- and microgenia is very common in Asians, and treatment requires a similar dosage to the 4–10 U recommended for Caucasians [27].

The use of botulinum toxin to reduce masseter width is well established in Asia as the most effective nonsurgical shaping and facial slimming tool [1, 3, 5, 6, 35, 36]. Asian women regard a square jaw as esthetically displeasing, and masseter reduction treatment is common in patients aged 18–30 years. In older patients, due to age-related facial volume loss and loss of skin elasticity, reducing the masseter volume can lead to worsening facial appearance, with hollowness and jowl formation. Therefore, treating their masseters becomes less of a priority.

The use of intradermal botulinum toxin or Microbotox [10, 11] is growing, as evidenced by the wide variation in the proportions of patients given this treatment between Asian countries where the treatment concept is still relatively new (China, Philippines, Thailand) versus those in which it has been used for many years (Singapore, Korea, Japan, Taiwan). Microbotox is the injection of multiple microdroplets of diluted onabotulinumtoxinA into the dermis of the skin or the interface between the dermis and the superficial layer of facial muscles that are attached to its undersurface [10, 11]. A reduction of sweat and sebaceous gland activity gives a smooth, lustrous texture to the skin, while the reduction of superficial facial muscle activity leads to a marked reduction of surface wrinkling. As the microdroplets are too small to diffuse into the deeper muscle, muscle function is retained, which gives a more natural, less frozen result. It is extremely useful in the forehead, where horizontal rhytides can be reduced without compromising eyebrow elevation and movement. It is also used to treat the glabellar and crow’s feet regions, as well as the lower face and neck (platysma). Less botulinum toxin is used with the Microbotox technique than with standard botulinum toxin injections. Typically, 20–28 U (0.5–0.7 ml of a 100 U botulinum toxin vial diluted with 2.5 ml saline) is drawn into a 1-ml syringe and topped up with 0.5–0.3 ml of 0.5 % lidocaine, to make a total volume of 1 ml of solution. To treat the entire forehead, 7-point glabellar and 8-point crow’s feet areas, and 1 ml of solution containing 20 U of botulinum toxin are sufficient. In the lower face and neck, 1 ml of solution containing 28 U is used per side (total of 56 U) to reduce superficial platysma activity and achieve better cervicomental and jawline contouring. The results of a Microbotox treatment can last for 3–4 months.

The proportion of Asian patients treated for volume or structural deficits in the lower face, and the types of treatments used, is shown in Table 8. In the Asian lower face, HA fillers are most commonly used in the chin (vs. the lips in Caucasians) [22]. Chin hypoplasia and mentalis hyperactivity are best treated with a combination of botulinum toxin and fillers [1]. Asians tend to have full lips; the top lip may be thicker than the lower lip. The moderate proportion of patients who request HA fillers in their lips do so, not to increase their volume, but rather to create better shape and esthetic balance. The Expert Group agreed that the “golden ratio” Phi (1:1.618) [37] is not applicable to the size and proportion of the upper and lower Asian lips. In Asians, while lips where the lower lip is larger than the upper by 1:1.618 are considered beautiful, not all Asian patients desire this relationship. Quite often Asians naturally have a larger upper lip in proportion to the lower lip and this helps offset an element of maxillary retrusion which is often seen in the Asian face. As such, most Asian patients prefer to have uniform enhancement of their lips and retain a relationship of upper to lower lip that may be the reverse of what is considered ideal in the Caucasian face.

### Consensus Statements for Lower Face Treatment

- A wide, square lower face is considered esthetically undesirable among Asian women, as reflected in the common request for treatment to reduce masseter muscle bulk.
- Chin retrusion is a common feature of the Asian face, which in turn leads to the high incidence of mentalis strain and hyperactivity of the mentalis muscle. This is reflected in the relatively high proportion of patients who are treated in the chin area with a combination of botulinum toxin and fillers.

## Conclusions

The use of nonsurgical esthetic facial treatments is increasing in Asia [38]. Surveys of current practice among this Expert Group showed that the types of treatment requested and provided correlate with anatomical structural deficiencies observed in the Asian face, as well as age-related changes, and they vary as patients age. Botulinum toxin and HA fillers are often used in combination. To achieve the desired esthetic outcome in younger Asian patients, addressing proportion is key. In older patients, maintaining facial structure and volume, in addition to addressing lines and folds, is essential to reducing the appearance of aging.

The desire for esthetic improvement, together with the complexity of the structural deficits and aging processes in the Asian face, often require a long-term management plan that involves multiple treatment modalities. The often wide variations in treatment practice and in the proportions of patients treated for particular indications (Tables 2, 3, 4, 5, 6, 7) are a reflection of different patient demographics, ethnicities, and “country-specific” popular desires; cultural differences; and varying injecting practices in the region, along with differences in perceptions of beauty that vary even among experts. In addition to a thorough understanding of the characteristic anatomy and morphology of the Asian face, a full-face approach and combination treatment strategies involving energy-based devices, botulinum toxin, and HA fillers are often required to address the complexity of many Asian patients’ esthetic concerns.

## Appendix

The Asian Facial Aesthetics Expert Consensus Group also includes Drs. Danru Wang, China; Yan Wu, China; Rashmi Shetty, India; Vandana Chatrath, India; Chytra Anand, India; Akiko Imaizumi, Japan; Nobutaka Furuyama, Japan; Hideaki Sato, Japan; Greg J. Goodman, Australia; Peter H. L. Peng, Taiwan; and Marisa Pongprutthipan, Thailand.
